# The POSEIDON Criteria and Its Measure of Success Through the Eyes of Clinicians and Embryologists

**DOI:** 10.3389/fendo.2019.00814

**Published:** 2019-11-20

**Authors:** Sandro C. Esteves, Carlo Alviggi, Peter Humaidan, Robert Fischer, Claus Y. Andersen, Alessandro Conforti, Klaus Bühler, Sesh K. Sunkara, Nikolaos P. Polyzos, Daniela Galliano, Michael Grynberg, Hakan Yarali, Irem Y. Özbek, Matheus Roque, Lan N. Vuong, Manish Banker, Laura Rienzi, Alberto Vaiarelli, Danilo Cimadomo, Filippo M. Ubaldi

**Affiliations:** ^1^ANDROFERT, Andrology and Human Reproduction Clinic, Campinas, Brazil; ^2^Department of Neuroscience, Reproductive Science and Odontostomatology, University of Naples Federico II, Naples, Italy; ^3^Fertility Clinic Skive, Skive Regional Hospital, Skive, Denmark; ^4^Faculty of Health, Aarhus University, Aarhus, Denmark; ^5^Fertility Center Hamburg, Hamburg, Germany; ^6^Laboratory of Reproductive Biology, Faculty of Health and Medical Sciences, University Hospital of Copenhagen, Copenhagen, Denmark; ^7^Center for Gynecology, Endocrinology, and Reproductive Medicine, Ulm, Germany; ^8^Department of Gynaecology, Jena-University Hospital-Friedrich, Schiller University, Jena, Germany; ^9^Faculty of Life Sciences and Medicine, King's College London, London, United Kingdom; ^10^Dexeus University Hospital, Barcelona, Spain; ^11^Instituto Valenciano de Infertilidad, Rome, Italy; ^12^Service de Médecine de la Reproduction et Préservation de la Fertilité, Hôpital Antoine Béclère, Clamart, France; ^13^Anatolia IVF, Ankara, Turkey; ^14^ORIGEN, Center for Reproductive Medicine, Rio de Janeiro, Brazil; ^15^Department of Obstetrics and Gynecology, University of Medicine and Pharmacy, Ho Chi Minh City, Vietnam; ^16^IVFMD, My Duc Hospital, Ho Chi Minh City, Vietnam; ^17^Nova IVI Fertility, Ahmedabad, India; ^18^GENERA, Center for Reproductive Medicine, Rome, Italy

**Keywords:** POSEIDON criteria, ovarian stimulation, low prognosis, poor ovarian response, oocyte, blastocyst, assisted reproductive technology, ART calculator

## Abstract

This article represents a viewpoint on the POSEIDON criteria by a group of clinicians and embryologists. Its primary objective is to contextualize the Poseidon criteria and their metric of success for the relevant Frontiers Research Topic “POSEIDON's Stratification of Low Prognosis Patients in ART: The WHY, the WHAT, and the HOW”. “Low prognosis” relates with reduced oocyte number, which can be associated with low or sometimes a normal ovarian reserve and is aggravated by advanced female age. These aspects will ultimately affect the number of embryos generated and consequently, the cumulative live birth rate. The novel system relies on female age, ovarian reserve markers, ovarian sensitivity to exogenous gonadotropin, and the number of oocytes retrieved, which will both identify the patients with low prognosis and stratify such patients into one of four groups of women with “expected” or “unexpected” impaired ovarian response to exogenous gonadotropin stimulation. Furthermore, the POSEIDON group introduced a new measure of clinical success in ART, namely, the ability to retrieve the number of oocytes needed to obtain at least one euploid blastocyst for transfer in each patient. Using the POSEIDON criteria, the clinician can firstly identify and classify patients who have low prognosis in ART, and secondly, aim at designing an individualized treatment plan to maximize the chances of achieving the POSEIDON measure of success in each of the four low prognosis groups. The novel POSEIDON classification system is anticipated to improve counseling and management of low prognosis patients undergoing ART, with an expected positive effect on reproductive success and a reduction in the time to live birth.

## Current Scenario

The proportion of patients of advanced female age and low ovarian reserve seeking fertility treatment is increasing worldwide. It is well-known that pregnancy rates are lower in these women than in younger counterparts. However, it is also important to realize that repetition of assisted reproductive technology (ART) treatments using a “trial and error” approach does not seem to help these patients, since the gap between older and younger patients, as regards cumulative pregnancy rates, increases after multiple IVF cycles (1).

In the era of personalized medicine, success in ART goes far beyond pregnancy. It should be redefined considering other quality dimensions, without overlooking the patient perspective (2–4). We believe that provision of proper evaluation, counseling about the chances of success, and development of an effective and safe time-limited treatment plan taking into full consideration the patients' values and preferences should be the cornerstones of healthcare delivered to infertile couples undergoing ART.

As far as evaluation is concerned, ovarian reserve biomarkers, like anti-Müllerian hormone (AMH) and antral follicle count (AFC), are now widely used to predict ovarian response to gonadotropin stimulation. Despite their clinical utility in this regard, the value of ovarian reserve biomarkers to predict reproductive success in ART is suboptimal (5–7). Furthermore, ovarian reserve markers cannot identify the hypo-responder patient, a concept firstly introduced by the Evian Annual Reproduction (EVAR) Workshop Group in 2008. These women, who differ from Bologna criteria poor responders in terms of age and ovarian reserve, have a stagnant response to exogenous FSH during ovarian stimulation and might end up having an unexpected poor or a suboptimal number of retrieved oocytes after conventional ovarian stimulation (8, 9).

By contrast, what became clear over the last years is a strong positive association between oocyte number and live birth rates (10–13). Nevertheless, the oocyte number should be combined with female age since the likelihood of achieving a live birth among patients with similar oocyte yield ultimately depends on the age of the patient (10). It means that the number of oocytes needed to maximize live birth should be individualized considering the age of the patient, and more importantly, patient-oriented strategies should be used to achieve the estimated individualized oocyte number.

## The Poseidon Criteria of “Low Prognosis” Patients Undergoing ART

**Figure 1:**
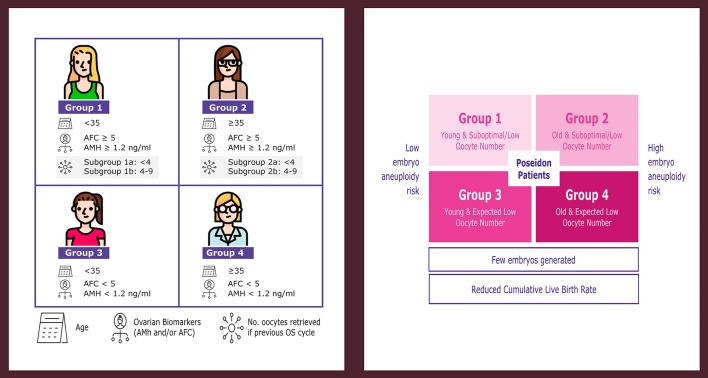
POSEIDON criteria of low prognosis patients in ART. The novel system relies on female age, ovarian reserve markers, ovarian sensitivity to exogenous gonadotropin, and the number of oocytes retrieved, which will both identify the patients with low prognosis and stratify such patients into one of four groups of women with “expected” or “unexpected” impaired ovarian response to exogenous gonadotropin stimulation. According to these criteria, four distinct groups of low prognosis patients can be established **(left)**. Owing to low oocyte numbers and less embryos produced, POSEIDON patients have lower cumulative live birth rates per started cycle than non-POSEIDON counterparts. However, the prognosis is differentially affected by oocyte quantity and female age, as the latter relates to the risk of embryo aneuploidy **(right)**. Art drawing by Chloé Xilinas. Modified from Esteves et al. (16). This is an open-access article distributed under the terms of the Creative Commons Attribution License (CC BY).

The issues mentioned above constitute the cornerstones of the novel POSEIDON (**P**atient-**O**riented **S**trategies **E**ncompassing **I**ndividualize**D O**ocyte **N**umber) criteria for “low prognosis” patients undergoing ART (14–17) (www.groupposeidon.com). The POSEIDON criteria propose a shift from the terminology of poor ovarian response (POR) to the concept of low prognosis. The low prognosis patient is classified into four groups according to the results of ovarian reserve markers (AMH, AFC, or both), female age, and the number of oocytes retrieved in previous cycles of conventional ovarian stimulation (OS)—in cases where this information is available (Figure 1). Patients fitting the POSEIDON criteria have low prognosis in ART owing to a decreased number of oocytes, which will limit the number of embryos produced. This condition might be aggravated further by advanced female age, thus negatively impacting the availability of genetically normal embryos for transfer, ultimately affecting the cumulative live birth rates (CLBR) per started cycle (13, 18).

Hence, the “low prognosis” concept fundamentally relates to cumulative live birth delivery rate, which is defined by the International Committee for Monitoring Assisted Reproductive Technologies (ICMART) (19) as, “*the number of deliveries with at least one live birth resulting from one initiated or aspirated ART cycle, including all cycles in which fresh and/or frozen embryos are transferred, until one delivery with a live birth occurs or until all embryos are used, whichever occurs first, expressed per 100 cycles (initiated or aspirated)*.”

**Figure 2:**
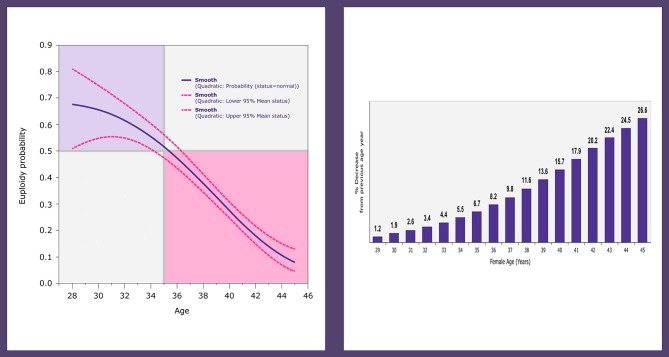
Logistic regression analysis of 1,220 trophectoderm biopsies from 436 patients undergoing ICSI and PGT-A by NGS. The plot depicts the fitted probabilities (with 95% confidence intervals) of blastocyst euploidy as a function of female age **(left)**. The graph shows the percent decrease in the probability of a blastocyst being euploid, which increases progressively with every year of female age **(right)**. Reprinted with permission of Edizioni Minerva Medica from Esteves et al. (21).

According to the POSEIDON criteria, the patients are classified as groups 1 and 3 if younger than 35 years old, and as groups 2 and 4 if older than 35 years of age (14–16). Female age is a critical element in the POSEIDON classification because age is crucially related to embryo ploidy and more importantly live birth outcome. In a study by the POSEIDON group involving infertile patients subjected to IVF-ICSI and pre-implantation genetic testing for aneuploidy (PGT-A) by next-generation sequencing analysis (NGS), the blastocyst euploidy probabilities were calculated as a function of female age (20). The probabilities mentioned above sharply declined after the age of 34 and were overall lower than 50% in women aged 35 years of age and over (Figure 2). This biological phenomenon, in combination with the already reduced ovarian reserve in patients with advanced female age, might increase the risk of having no euploid embryos for transfer (20). In the above study, the percent decline in blastocyst euploid probability increased progressively with advancing female age. The geometric mean of the yearly variation was 13.6%. However, it increased progressively year on year. At age 30 it was 2.0%, whereas, at ages 35, 39, and 44, the relative loss in the blastocyst euploidy probabilities were 6.7, 13.6, and 24.5%, respectively (Figure 2). These figures indicate that the older the patient, the higher the number of oocytes and embryos needed to increase the chances of having at least one euploid blastocyst within the cohort of embryos (20).

Collectively, patients fitting POSEIDON's groups 1 and 3 are young and, therefore, the risk of embryo aneuploidy is relatively low. By contrast, groups 2 and 4 include older patients with an increased risk of embryo aneuploidy (Figure 1). As a result, irrespective of the group, the number of embryos generated would be likely low, thus affecting the CLBR per started cycle. Importantly, despite having an overall low prognosis, the CLBR per started cycle will differ according to the classification group as it is affected by female age and oocyte number.

## Clinical Validation Data

Leijdekkers et al., in 2019, used the data from the OPTIMIST prospective study to assess the CLBR in low-prognosis patients stratified according to the POSEIDON criteria (22). The authors showed that the prognosis concerning CLBR differed among the low-prognosis groups, with maternal age (and hence oocyte quality) being the dominant determinant of CLBR rather than the quantitative ovarian reserve. Thus, the authors concluded that given the fact that the differences in CLBR among POSEIDON groups are primarily due to the effect of maternal age on oocyte quality, the new criteria have limited value for clinical management, although it might be used for patient counseling.

Having scrutinized the authors' data, we found that in addition to confirming what clinicians already know about the primary role of maternal age on the likelihood of achieving a live birth in ART, the study of Leijdekkers et al. also confirm that CLBR is affected not only by age but definitely by the number of oocytes retrieved (22). In their study, the authors showed that the CLBR in low-prognosis patients was ~56% over 18 months follow-up. Notably, the CLBR was surprisingly high in all POSEIDON groups, reaching ~68 and 39% in Poseidon groups 1b and 4, respectively, as compared to 72 and 58% in younger and older non-POSEIDON patients. However, such figures were achieved after an average of two fresh transfer cycles per woman, which is not in line with the CLBR definition by the ICMART (19). It is important to realize that the concept of low-prognosis introduced by the POSEIDON group concerns CLBR per started cycle, as defined by the ICMART (16). By contrast, the per-period estimation might inflate CLBRs owing to the high dropout rate after the first failed IVF treatment (23).

Indeed, when Leijdekkers et al. (22) evaluated CLBRs per cycle, there was a remarkable difference between POSEIDON patients (21, 43, 10, 25, 29, and 17% in groups 1a, 1b, 2a, 2b, 3, and 4, respectively) and non-POSEIDON counterparts (52%). Moreover, their data show that CLBR per cycle was twice as high in patients with a suboptimal response to stimulation (4–9 oocytes) compared to those with a low response to stimulation (<4 oocytes) both in women <35 years-old (group 1b: 43%; group 1a: 21%) and ≥35 years-old (group 2b: 25%; group 2a: 10%). Not surprisingly, the CLBRs per cycle in young (29%) and old (17%) expected poor responders (POSEIDON groups 3 and 4, respectively) were similar to those of POSEIDON groups 1a and 2a. These figures have clinical importance because CLBR per started cycle might be increased in low prognosis women by increasing the number of retrieved oocytes, which may be achieved mainly in POSEIDON patients who have adequate ovarian reserve markers, that is, groups 1 and 2 (8, 17, 18, 24, 25).

Thus, in addition to serve as a counseling tool, we suggest that the POSEIDON criteria should be used to guide clinical management with a specific focus on optimizing the follicle:oocyte ratio (FOI) to achieve higher reproductive outcomes. In patients with an unexpected poor/suboptimal oocyte number due to a low FOI (e.g., groups 1 and 2), it has been suggested that individualization of ovarian stimulation might increase the number of oocytes retrieved (17, 18). However, patients with an expected low oocyte number could also benefit from individualized regimens, in which pharmacological interventions should be combined with oocyte/embryo accumulation (26, 27).

## The Rationale of Individualizing the Oocyte Number

Big data indicates that there is a positive association between the number of oocytes and CLBR per started cycle, with higher oocyte thresholds for better outcomes (13, 18). Although this information gives the clinician some guidance, high oocyte numbers might be hard to achieve in POSEIDON patients. Thus, the POSEIDON group introduced a new metric of success in ART, namely, *the ability to retrieve the number of oocytes needed to obtain at least one euploid blastocyst for transfer in each patient* (14, 15). The POSEIDON marker of success seems to be a logical endpoint for clinicians providing care to women undergoing ART because the transfer of a euploid embryo provides –at any given age– implantation rates in the range of 50–60% overall (28). Importantly, this endpoint does not imply that PGT-A should be routinely performed during ART.

We acknowledge that live birth rate (LBR) is the primary endpoint for couples undergoing ART (29). Nonetheless, LBR has been reported in only a small proportion of studies and depends on a multitude of controlled and uncontrolled factors, thus making it challenging to use LBR for making individualized predictions about the number of oocytes needed to achieve the desired outcome. In particular, LBRs in low responders and advanced age women are influenced by the age-dependent miscarriage rate observed in these subgroups. For instance, the miscarriage rate in women over 40 years was estimated to be ~30% (30). Not surprisingly, a dramatic drop-out before delivery is observed during trials. Moreover, LBR is prone to biases not related to ART. For instance, intrauterine fetal death after 12 weeks of gestation occurs in about 5% of ongoing pregnancies, whose risk further increases in women of advanced age (31).

Hence, other endpoints, such as the one proposed by the POSEIDON group, might be considered as we feel it is essential to acknowledge the continuum of reproductive outcomes like implantation rates, pregnancy rates, clinical pregnancy rates, ongoing pregnancy rates, and LBR. Naturally, infertility is a couple's problem, and a single intermediate metric (such as the one introduced by the POSEIDON group) is limited to predict treatment outcome. Thus, we are not suggesting that LBR should be replaced by the new metric but do believe it adds independent information that may allow for better treatment planning. The clinician can objectively estimate the individualized oocyte number to achieve at least one euploid embryo for transfer by either looking at the embryonic data of her/his particular clinic or using predictive models.

## The ART Calculator

Recently, a new predictive tool, called the “ART Calculator,” was developed to estimate the minimum number of metaphase II (MII) oocytes required to have at least one euploid blastocyst for transfer in patients undergoing ART (21). To achieve this goal, firstly, there was a search for relevant predictors. The observational unit and the response variable were respectively (i) the woman, and (ii) the pair (*m, n*), where *n* is the number of retrieved metaphase II oocytes and m the corresponding number of euploid blastocysts. A penalized regression model, with the negative binomial for the distribution of euploid blastocysts and the log link function, was used for the selection of predictors. The negative binomial was chosen from first principles and from the heuristic fact that this distribution fitted the data very closely. The selection of predictors was carried out by the Lasso method, a procedure that allows for the fitting of correlated and high-dimensional data. Among 26 predictors tested from ~350 infertile couples undergoing IVF/ICSI and PGT-A, female age, and type of sperm used for IVF/ICSI were found to be the relevant predictors concerning blastocyst euploidy.

The final predictive model provides the age-related probabilities of a blastocyst being euploid per metaphase II (MII) oocyte as a function of sperm type (ejaculated, epididymal, or testicular sperm, and adjusted for the type of azoospermia, that is, obstructive or non-obstructive azoospermia). The data indicated that the estimated probability of an MII oocyte turn into a euploid blastocyst decreases progressively with female age, an effect that is negatively modulated by the use of testicular sperm from men with non-obstructive azoospermia (NOA) (21). The above results are consistent with previous reports. Indeed, with aging, oocyte chromosomal abnormalities and cytoplasmic dysfunctions increase, whereas the number of primordial follicles progressively decline (20, 32–35). Moreover, the use of testicular sperm from men with NOA was shown to be a negative predictor for obtaining a euploid blastocyst per oocyte pickup, most probably related to the fact that the blastocyst rate per fertilized oocyte is significantly reduced (21, 36).

Using the probabilities mentioned above and mathematical equations, the ART calculator provides individualized estimations about the minimum number of MII oocytes required to obtain at least one euploid blastocyst, with 95% confidence interval [CI]. Specifically, the ART calculator makes two types of predictions automatically, one using pretreatment information to estimate the minimum number of MII oocytes to achieve at least one euploid blastocyst, and another based on the actual number of mature oocytes collected/accumulated to estimate the chances of having a euploid blastocyst using that oocyte cohort for IVF/ICSI (http://www.members.groupposeidon.com/Calculator/).

As an example, a hypothetical couple undergoing IVF/ICSI whose female partner is 36 years old and the male partner has moderate oligoasthenoteratozoospermia—thus ejaculated sperm will be used for sperm injections—needs at least nine metaphase II oocytes (confidence interval: 7–10) to obtain at least one euploid blastocyst for transfer, considering a 80% probability of success (set by the user) (source: http://www.members.groupposeidon.com/Calculator/). Let us now consider that the patient under discussion belongs to POSEIDON's group 2. Using the POSEIDON criteria and the ART calculator, the treating physician can plan the ovarian stimulation strategy with the mindset of optimizing the FOI to achieve the predicted number of metaphase II oocytes or higher (17, 25). If the target oocyte number is achieved, the exemplary couple's chance of having at least one euploid blastocyst for transfer in the resulting embryo cohort will be 80% (or 20% risk of failure). It is well-known that single euploid blastocyst transfer gives ~50–60% implantation rates (30). Thus, given the risk of spontaneous miscarriage and intrauterine fetal death after 12 weeks of gestation of about 10%, the ultimate live birth rate for the hypothetical couple will be about 40% (28). These figures are remarkably higher than the LBR of ~30% reported for such couples without the “POSEIDON's approach” (37). On the other hand, if the exemplary couple belonged to Poseidon's group 4 and the number of retrieved metaphase II oocytes were four after the above exercise, the revised estimates would indicate a ~51% probability of having at least one euploid blastocyst with that oocyte number (source: http://www.members.groupposeidon.com/Calculator/). In this scenario, the health care provider and the affected patients could decide the best way to move forward, which might include, for instance, going ahead with fertilization, embryo culture and transfer (with or without PGT-A), or exploring oocyte/embryo accumulation (27).

Detailed information about the calculator development is available in a dedicated article within this Frontiers Research Topic (21). Although other female factors, such as obesity, ethnicity, previous pregnancy, infertility etiology, and ovarian reserve markers are important for ovarian stimulation success, they were not deemed informative for the ART calculator predictive model, which used blastocyst euploidy per MII oocyte as the response. However, it is worth mentioning that there was no attempt to determine fundamental associations between the predictors and the number of euploid blastocysts. Along these lines, “power” is not a relevant concept in predictive modeling nor are sequential temporal associations concerning the ability of an MII oocyte turn into a euploid blastocyst. The primary objective of the ART calculator study was the development of a prediction formula for the number of euploid blastocysts. The resulting model was subjected to validation by the holdout sample method. The quality of the predictive model was assessed by the ROC curve, calculated on the holdout sample. The predictive ability of the model assessed by the area under the ROC curve was ~72%, thus suggesting that unknown factors intrinsically related to the biological variability of oocytes and embryos might also influence their ploidy status (21).

From both clinical and embryological perspectives, the ART calculator provides objective information, which might help patients prepare themselves both emotionally and financially for the treatment journey. Moreover, the ART calculator provides clinicians an estimation of the minimum number of mature oocytes required for at least one euploid blastocyst in IVF/ICSI procedures, which improves the planning of the specific treatment. Nonetheless, clinicians should not deny treatment to infertile women if the predicted number of oocytes needed to achieve at euploid blastocyst is too high or the probabilities of achieving this goal—based on the actual number of oocytes retrieved—is too low. The embryos are statistically independent concerning the ploidy status, which primarily depends on maternal age (31). Thus, the euploid embryo could be anywhere within the patient embryo cohorts.

## Patient-Oriented Strategies to Achieve the Individualized Oocyte Number

Using the POSEIDON criteria, the clinician can, first of all, identify and classify patients who are likely to have reduced success in ART, and secondly, develop a treatment plan to achieve the individualized oocyte number related to the optimal probability of generating at least one euploid blastocyst for transfer in each POSEIDON's patient category.

In practical terms, the individualized oocyte number can be achieved using patient-oriented strategies. For instance, the type of GnRH analog, type of gonadotropin, the starting dose, and the regimen may be tailored according to POSEIDON stratification (8, 38–41). Importantly, patient-oriented gonadotropin dosing aimed at retrieving more oocytes does not seem to affect the embryo ploidy status. In an ongoing multicenter study by the POSEIDON group, we observed that the age-controlled probability of a blastocyst being euploid is not affected by the size of embryo cohort (unpublished data), thus confirming previous observations of a lack of detrimental effect on embryo ploidy in patients who had more oocytes retrieved (20, 32). Our observations also indicate that the use of minimal or mild stimulation –as compared to conventional stimulation– has no apparent positive effect on embryo genetic competence. What matters most concerning embryo ploidy is female age and not the intensity of ovarian stimulation (42–45).

In reality, low gonadotropin dosing or suboptimal gonadotropin regimen might result in hypo-response and the retrieval of fewer than expected oocytes (8, 16, 18, 24, 40). This phenomenon can be better appreciated in POSEIDON groups 1 and 2, who despite adequate pre-stimulation ovarian parameters end up having a poor or suboptimal oocyte yield, possibly due to inappropriate gonadotropin dosing/regimen and/or the presence of genetic polymorphisms affecting the gonadotropins and their receptors (9, 17, 25, 46). Therefore, a thorough evaluation of the patient is critical to help the clinician identify the low prognosis patient and plan a treatment tailored to the patient's specific needs. It has been suggested that individualization of ovarian stimulation might increase the number of oocytes retrieved among patients with an unexpected poor/suboptimal oocyte number (POSEIDON's groups 1 and 2), in particular, those with a low FOI (8, 9, 24). Naturally, the use of the right gonadotropin starting dose and the possibility to adapt the dose and the regimen during the cycle is essential to optimize oocytes yield while securing patient safety (18, 47–50).

Notwithstanding, even using the best protocol, the individualized oocyte number might be difficult to achieve with a single ovarian stimulation. This observation is particularly relevant for patients in POSEIDON's groups 3 and 4, who all have a reduced ovarian reserve. In such cases, treatment should be planned with the mindset that the number of oocytes needed to achieve at least one euploid blastocyst is lower in young (group 3) than in older (group 4) patients (21). Individualized regimens, possibly combining pharmacological interventions and oocyte/embryo accumulation, could also benefit these patients as a means of shortening the time frame to reach the target oocyte number (26, 27, 41, 48–54).

## Future Directions

The critical data necessary to support the clinical uptake of the POSEIDON criteria would involve the confirmation that (i) patients fitting the four groups have low prognosis as compared to non-POSEIDON patients concerning the CLBR per started cycle, and (ii) patient-oriented strategies with the mindset to achieve the POSEIDON's measure of success increase the continuum of reproductive outcomes, including the time to live birth. The patient population characteristics, discovery set, and the independent validation steps for building and confirming the associative success of the POSEIDON classification are ongoing, and the first results have been recently published (22, 26, 55, 56). While awaiting the results of randomized trials to clarify the role of interventions in this vast and important group of ART patients, we would suggest that individualization of the ovarian stimulation is superior to a “one size fits all” policy in POSEIDON patients.

## Conclusions

The novel POSEIDON classification of the low prognosis patient in ART combined with the use of patient-oriented strategies to achieve the individualized oocyte number—as predicted by the ART calculator—should be considered by clinicians to reduce the time to live birth. This new system may help improve patient counseling and management, with an expected positive effect on IVF success and time to live birth. We invite readers to learn more about the POSEIDON initiative and the ART Calculator at both www.groupposeidon.com and this Frontiers Research Topic https://www.frontiersin.org/research-topics/6849/poseidons-stratification-of-low-prognosis-patients-in-art-the-why-the-what-and-the-how. The POSEIDON group is an open access initiative; thus, we encourage our colleagues to join us as POSEIDON members (please find out more at http://www.groupposeidon.com/member-benefits/).

## Data Availability Statement

The datasets generated for this study are available on request to the corresponding author.

## Author Contributions

SE and PH contributed to the conception and designed the manuscript. SE wrote the first draft of the manuscript. CA, PH, RF, CYA, AC, KB, SS, NP, DG, MG, HY, IÖ, MR, LV, MB, LR, AV, DC, and FU wrote sections of the manuscript. All authors contributed to manuscript revision, read and approved the submitted version.

### Conflict of Interest

SE declares the receipt of unrestricted research grants from Merck, and lecture fees from Merck, Besins, Gedeon-Richter, and Lilly. PH has received unrestricted research grants from MSD, Merck, and Ferring as well as honoraria for lectures from MSD, Merck, Gedeon–Richter, Theramex, and IBSA. CA, RF, and MR have received honoraria for lectures from Merck. CYA has received unrestricted grants from Gedeon-Richter and honoraria for lectures from IBSA, Ferring, and Merck. KB has received honoraria for lectures from Takeda, Ferring, and Merck. SS and MB declare the receipt of honorarium for lectures from Merck, MSD, and Ferring. NP has received unrestricted grants or lecture fees from Merck, MSD, Gedeon-Richter, IBSA, Ferring Pharmaceuticals, and Theramex. MG declares receipt of lecture fees from Merck, Gedeon-Richter, Ferring, Roche, and Ipsen. HY declares receipt of honorarium for lectures from Merck and Ferring. LV has received speaker's fees from Merck, Ferring, and research grants from MSD. FU, LR, and AV have received honoraria for lectures from MSD and Merck. The remaining authors declare that the research was conducted in the absence of any commercial or financial relationships that could be construed as a potential conflict of interest.
